# Seroprevalence and Risk Factors Analysis of Foot‐and‐Mouth Disease in Cattle and Buffaloes in Punjab of Pakistan: A 3ABC ELISA‐Based Study

**DOI:** 10.1155/tbed/9504319

**Published:** 2026-11-03

**Authors:** Rai Bahadur Kharl, Mughees Aizaz Alvi, Hamza Samee, Xusheng Ma, Zixiang Zhu, Ali Zohaib, Aliza Ali, Muhammad Zeeshan, Fan Yang, Wen Dang, Jijun He, Hong-Bin Yan, Muhammad Saqib, Haixue Zheng

**Affiliations:** ^1^ State Key Laboratory for Animal Disease Control and Prevention/College of Veterinary Medicine Lanzhou University/WOAH Reference Laboratory for Foot-and-Mouth Disease /National Reference Laboratory for Foot-and-Mouth Disease/Gansu Province Research Center for Basic Disciplines of Pathogen Biology/Key Laboratory of Veterinary Etiological Biology and Key Laboratory of Ruminant Disease Prevention and Control (West) Ministry of Agricultural and Rural Affairs Lanzhou Veterinary Research Institute Chinese Academy of Agricultural Sciences Lanzhou 730046 China caas.cn; ^2^ Department of Microbiology College of Veterinary Medicine King Faisal University Al-Ahsa 31982 Saudi Arabia kfu.edu.sa; ^3^ One Health Research and Innovation Center (OHRIC) King Faisal University Al-Ahsa 31982 Saudi Arabia kfu.edu.sa; ^4^ Department of Clinical Medicine and Surgery University of Agriculture Faisalabad Pakistan uaf.edu.pk; ^5^ Infectious Disease Research Department King Abdullah International Medical Research Center Jeddah Saudi Arabia kaimrc.med.sa; ^6^ King Saud bin Abdulaziz University for Health Sciences Jeddah Saudi Arabia ksau-hs.edu.sa; ^7^ Ministry of the National Guard–Health Affairs Jeddah Saudi Arabia; ^8^ Department of Parasitology University of Agriculture Faisalabad Pakistan uaf.edu.pk; ^9^ Department of Pathobiology Riphah College of Veterinary Sciences Riphah International University Lahore Pakistan riphah.edu.pk

**Keywords:** 3ABC ELISA, foot-and-mouth disease, Pakistan, Punjab, risk factors, seroprevalence

## Abstract

Foot‐and‐mouth disease (FMD) is an important transboundary livestock disease that can cause significant economic losses in endemic areas. This study aimed to assess the seroprevalence of antibodies against the nonstructural proteins (NSPs) of FMD virus (FMDV) and associated animal‐ and farm‐level risk factors in cattle and buffaloes of nine livestock‐producing districts in Punjab, Pakistan. A 6‐month field survey was conducted in which a total of 576 animals (322 cattle and 254 buffaloes) were sampled, and a 3ABC ELISA was used. The assay measures antibodies to FMDV NSPs, supporting a DIVA (differentiation of infected from vaccinated animals) strategy, as NSP antibodies are more characteristic of viral replication than vaccination with purified inactivated vaccines. However, vaccination histories were not available for the sampled animals, and NSP seropositivity in this study was regarded as indicative of prior FMDV exposure, with the possibility of residual NSPs in incompletely purified vaccines acknowledged. Overall FMDV NSPs seropositivity was 71.18% (410/576), ranging from 33.33% in Kasur to 89.36% in Faisalabad. The NSP seropositivity was higher in cattle (75.16%) than in buffaloes (66.14%), but the difference was not statistically significant (odds ratio [OR] = 1.14, 95% confidence interval [CI]: 0.88–1.47; *p* = 0.3289). The odds of seropositivity were higher in female animals than in male animals (74.72% vs. 26.19%; OR = 2.85, 95% CI: 1.46–5.58; *p* = 0.0016), but this should be interpreted with caution because of the highly skewed sex distribution. Animals over 10 years old were more likely to be FMDV NSPs seropositive than animals ≤5 years old (OR = 1.96, 95% CI: 1.24–3.08; *p* = 0.0085). Cattle–sheep mixed‐species contact was also associated with higher NSP seropositivity (90.91%; OR = 2.73, 95% CI: 1.53–4.85; *p* = 0.0417). These results suggest significant FMDV exposure in the sampled livestock population and emphasize the need for risk‐based surveillance, farm animal‐movement management, and enhanced farm biosecurity for FMD control in Punjab.

## 1. Introduction

Cattle and buffaloes are cloven‐hooved animals that are susceptible to foot‐and‐mouth disease (FMD), a highly infectious viral disease that causes huge economic losses [1, 2]. The World Organization for Animal Health (WOAH) considers FMD a disease subject to mandatory notification [3]. There are seven unique serotypes of FMD virus (FMDV), a member of the family Picornaviridae, that cause FMD: O, A, C, SAT 1, SAT 2, SAT 3, and Asia 1 [4, 5]. Researchers face significant challenges in developing effective vaccines and controlling FMD because the virus shows high genetic variability and rapid antigenic changes [6–8].

FMD control remains challenging in Pakistan, along with other South Asian countries, as the disease remains endemic and outbreaks occur frequently due to insufficient vaccines, undiagnosed cases, and free movement of animals [9–14]. FMD remains a major concern across Punjab province, a major cattle and buffalo farming hub, particularly in traditional rural regions where animals graze together under traditional agricultural practices [5, 15]. In Pakistan, FMD control relies on inactivated vaccines, including locally produced oil‐adjuvanted vaccines and imported purified vaccines.

Serological surveys measure FMDV antibody presence to evaluate animal population exposure and immunological status, enabling an understanding of FMD epidemiology [8]. The 3ABC ELISA (enzyme‐linked immunosorbent test) detects nonstructural protein (NSP) among viral proteins to differentiate infected from vaccinated animals using DIVA methodology [16, 17]. This testing method effectively identifies asymptomatic carriers and evaluates immunization campaigns in endemic regions that conduct mass vaccinations [18]. Inactivated vaccines, along with surveillance, play an important role in FMD in endemic countries like Pakistan. Antibodies against NSPs develop after viral replication and are thus widely used to detect prior FMDV infection and to support the differentiation of infected from vaccinated animals (DIVA), especially when purified vaccines are used. Interpretation of NSP serology should take into account the type and quality of the vaccines applied in the region since some products may, in rare cases, produce NSP antibodies as a result of residual NSPs in incompletely purified vaccines [2, 19].

The circulation of three primary serotypes, namely, O, A, and Asia 1, enumerated in previous research in Pakistan, raises significant hurdles for disease control as well as vaccine effectiveness [20, 21]. This study applies the 3ABC ELISA to measure FMD seropositivity rates among cattle and buffaloes across the Punjab province, Pakistan. The research investigates FMD seropositivity‐linked risk factors, including species, age demographics, sex, contact type, and farm environment, across nine different districts of Punjab province. The findings will enhance FMD epidemiology in the region and provide essential information for developing targeted control strategies.

## 2. Materials and Methods

### 2.1. Research Area

The study was conducted at cattle and buffalo farms in Faisalabad, Okara, Bahawalpur, Bahawalnagar, Kasur, Rahim Yar Khan, Khushab, Bhakkar, and Khanewal, all within Pakistani territory (Figure 1). These districts act as vital business centers that support the national livestock industry, contributing a large proportion of national meat and dairy products. The region’s extensive farming diversification, with farm operations ranging from large‐scale to semi‐intensive to intensive livestock systems, makes the study areas suitable to investigate FMDV NSP seroprevalence and risk factors. Furthermore, FMD epidemiology may change with seasonal patterns and animal migration dynamics in regions with dry to semiarid climates.

**Figure 1:**
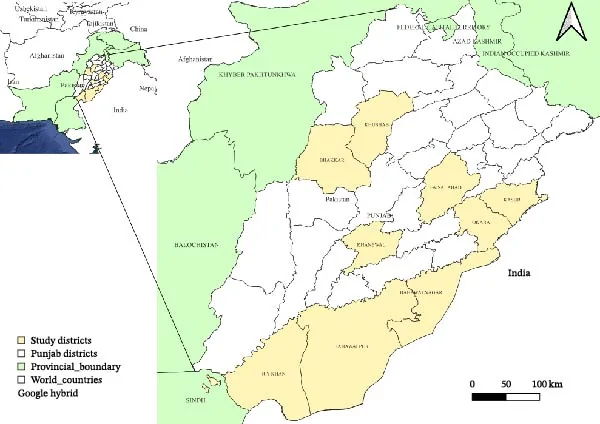
The study regions chosen to collect blood samples from cattle and buffaloes across nine major livestock‐producing districts in Pakistan.

### 2.2. Study Population and Sampling Strategy

The research analysis included cattle and buffaloes, regardless of age or sex. The study did not collect FMDV vaccination history for the sampled animals because farmers were unwilling to provide accurate information. A convenience sampling approach was adopted. Eligible cattle and buffaloes at the selected farms during the study period were included after obtaining the owner’s consent. The minimum sample size was calculated according to the formula described by Thrusfield [22]:

n=Z2P1−P/d2,

where *n* is the required sample size, *Z* = 1.96 for a 95% confidence level, *P* is the expected prevalence (50% as reliable prevalence estimates were not available in all selected districts), and *d* is the precision desired (5%). We calculated a sample size of 384 animals. A total of 576 animals (322 cattle and 254 buffaloes) were ultimately included to improve geographical representation and increase statistical power.

### 2.3. Inclusion and Exclusion Criteria

Animals older than 6 months were included in the study. Apparently healthy animals kept under normal farm management, with the owners’ willingness to sample, were eligible.

Animals under 6 months of age, animals that were too sick, animals with incomplete epidemiological data, and samples showing hemolysis or an insufficient volume of serum were excluded.

### 2.4. Data Collection

A uniform questionnaire was designed to systematically collect data on both individual animal traits and farm management practices. Laboratory technicians collected animal demographic data, including age, sex, etc., to assess possible risk factors linked to FMD seropositivity. The researchers also collected information on other livestock contacts to determine how quickly the transmission progressed. The questionnaire also included information regarding farm environmental conditions. This investigation measured epidemiological data to determine their relation to FMD prevalence, as found in previous studies [23, 24].

### 2.5. Sample Collection and Processing

Sample collection was conducted over a period of 6 months across the selected districts. Sterile needles and vacutainer tubes were used to obtain blood by making jugular vein punctures on each animal. Five milliliters of blood per animal under proper animal care and minimal stress conditions were taken. The collected samples received unique identification numbers linked directly to the survey answer sets. The blood was collected at room temperature and allowed to clot before 10‐min centrifugation at 3000 rpm for serum extraction. The collected sera were transferred into Eppendorf tubes and stored at −20°C until the analysis became available. Standard cold‐chain procedures were used to ship samples and preserve their structural integrity.

### 2.6. Detection of Antibodies Using 3ABC ELISA

The study had no information on FMDV vaccination history for the animals sampled. This information was unavailable either because farm registers were incomplete or because farmers provided unreliable information about the vaccination status. In Punjab, Pakistan, the FMD control program has used both locally produced and imported inactivated vaccines, and vaccine purity varies from one to another. The presence of antibodies measured by the FMDV NSP 3ABC ELISA was therefore considered indicative of previous exposure to FMDV. Still, it should be interpreted in light of the possible residual NSP contamination in the vaccines. The ELISA plates containing the 3ABC antigen were run according to the manufacturer’s instructions for conducting the assay with serum samples. An automated ELISA reader (iMark, Bio‐Rad Laboratories, Hercules, CA, USA) measured the color development at 450 nm optical density (OD) after adding the secondary enzyme‐linked antibody to the solution. The researchers interpreted their findings using percentage inhibition (PI) measurements. Raw OD values measured at 450 nm were used only for exploratory statistical analyses of the assay signal distribution. We based diagnostic interpretation only on PI, as recommended by the manufacturer. Samples with a PI > 50% were classified as NSP antibody positive, while samples with a PI < 50% were classified as NSP antibody negative [17, 25]. The assay was valid only if the control values were within the manufacturer‐specified quality control range. Raw OD values were used only for exploratory comparisons of serological reactivity among groups and were not interpreted as direct measures of disease severity, viral load, or infection intensity.

### 2.7. Statistical Analysis

Data analysis was conducted in SPSS Version 25.0 (IBM Corp., USA) after data entry in Microsoft Excel. Descriptive statistics provided complete seroprevalence figures for FMDV NSP across the studied population. We used univariate and multivariable logistic regression analyses to identify risk factors for FMD seropositivity. The multivariable model included age, sex, species, type of contact, and farm environment as independent variables. Multicollinearity among the independent variables before fitting the model using variance inflation factors (VIFs) was checked. A VIF below 5 as the criterion for no problematic multicollinearity. Odds ratios (ORs) with 95% confidence intervals (CIs) were calculated. We evaluated model fit using the Hosmer–Lemeshow goodness‐of‐fit test. A *p*‐value greater than 0.05 was considered indicative of an adequate model fit.

## 3. Results

The seroprevalence of FMDV NSPs varied across districts from 33.33% to 89.36% (Figure 2). Seroprevalence was highest in Faisalabad (89.36%), followed by Bhakkar (88.19%) and Bahawalnagar (82.35%), while it was lowest in Kasur (33.33%).

**Figure 2:**
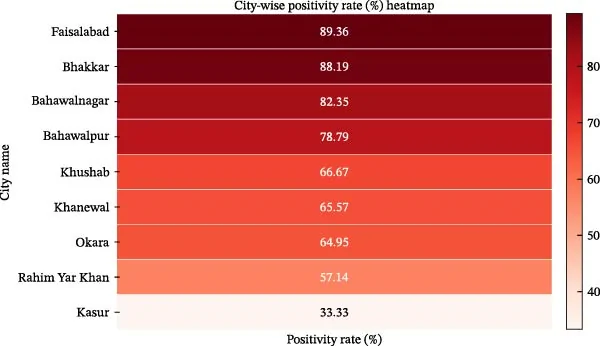
Geographic distribution of disease prevalence across study areas.

Univariate logistic regression revealed that animals from Faisalabad (OR = 2.68) and Bhakkar (OR = 2.65) had higher odds of being NSP seropositive than animals from Kasur. Still, these differences were not statistically significant (*χ* ^2^ = 14.99, *p* = 0.0594).

Although district‐wise seroprevalence did not differ significantly (*χ* ^2^ = 14.99, *p* = 0.0594), ANOVA demonstrated significant differences in the mean OD values among districts (*F* = 23.54, *p*  < 0.05). Tukey’s post hoc analysis showed significant differences between pairs of data, especially between Bhakkar and Kasur. Multicollinearity diagnostics showed no evidence of problematic collinearity among the principal predictors (age, sex, and species), with VIF values of 1.40, 1.42, and 3.39, respectively. The VIF for the farm environment type was also lower than 5. A higher VIF value was only found for the dummy variables created for the multicategorized contact‐type variable and are not necessarily problematic because the dummy coding was an expected result. The Hosmer–Lemeshow goodness‐of‐fit test showed that the final logistic regression model adequately fitted the observed data (*χ* ^2^ = 7.53; *p* = 0.481).

### 3.1. Species‐Wise Results

A higher seroprevalence of FMDV NSPs was observed in cattle than in buffaloes (75.16% vs. 66.14%), suggesting that cattle were more exposed to FMDV than buffaloes under the study conditions. Seroprevalence was higher among cattle, but the association between species and NSP seropositivity was not confirmed (OR = 1.14, 95% CI = 0.88–1.47; *p* = 0.3289). Mean OD values differed significantly between species (ANOVA, *F* = 23.41, *p*  < 0.001). Multivariable regression showed that buffaloes had significantly higher OD values than cattle (*p*  < 0.001), suggesting greater serological reactivity among seropositive buffaloes. After adjusting for age and sex in multivariable regression, buffaloes had significantly higher OD values than cattle (*p*  < 0.001). Male sex was also related to higher values of OD (*p*  < 0.001), and age was modestly associated (*p* = 0.024). The model accounted for 15.6% of the variance in OD values (*R* ^2^ = 0.156).

### 3.2. Sex‐Wise Results

The seroprevalence of FMDV NSPs was significantly higher among females (74.72%; 399/534) than males (26.19%; 11/42). Female animals had significantly greater odds of NSP seropositivity than males (OR = 2.85, 95% CI: 1.46–5.58; *p* = 0.0016). Mean OD values differed significantly between sexes (*t* = 5.87, *p*  < 0.001), with males having significantly higher means than females.

### 3.3. Age Group‐Based Results

The seroprevalence of FMDV NSPs increased with age. Animals ≤5 years had the lowest seroprevalence, while animals >5 years had a prevalence of more than 75% and the highest prevalence in animals >10 years. Seroprevalence was typically higher than 78% in the oldest age groups of animals, though sample sizes for these were relatively small. In comparison to animals ≤5 years of age, animals >10 years were significantly more likely to be seropositive for NSP (OR = 1.96, 95% CI: 1.24–3.08; *p* = 0.0085) and thus, age proved to be an important risk factor for NSP seropositivity (Table 1). Mean OD values differed significantly across all age groups (ANOVA: *F* = 19.03, *p*  < 0.001). Tukey’s post hoc analysis showed significantly higher OD in animals older than 10 years than in those ≤5 years, with no significant difference between the two older age categories.

**Table 1:** Univariate analysis of risk factors associated with disease prevalence.

Variable	Category	Positive/tested	Prevalence (95% CI)	Odds ratio (95% CI)	Chi‐square value (*p*‐value)
District	Faisalabad	42/47	89.36 (77.40–95.37)	2.68 (1.56–4.60)	14.99 (0.0594)
	Bhakkar	112/127	88.19 (81.43–92.71)	2.65 (1.52–4.60)	
	Bahawalnagar	28/34	82.35 (66.48–91.65)	2.47 (1.23–4.96)	
	Bhawalpur	52/66	78.78 (67.49–86.92)	2.36 (1.28–4.35)	
	Khusab	40/60	66.66 (54.06–77.27)	2.00 (1.06–3.76)	
	Khanwal	40/61	65.57 (53.05–76.25)	1.97 (1.05–3.70)	
	Okara	63/97	64.94 (55.05–73.71)	1.95 (1.09–3.49)	
	Rahim Yar Khan	12/21	57.14 (36.54–75.53)	1.71 (0.73–4.03)	
	Kasur	21/63	33.33 (22.95–45.63)	Reference	
Species	Cattle	242/322	75.16 (70.16–79.56)	1.14 (0.88–1.47)	0.95 (0.3289)
	Buffaloes	168/254	66.14 (60.12–71.68)	Reference	
Sex	Female	399/534	74.72 (70.87–78.22)	2.85 (1.46–5.58)	10.00 (0.0016)
	Male	11/42	26.19 (15.30–41.07)	Reference	
Age	>10 years	156/198	78.79 (72.57–83.91)	1.96 (1.24–3.08)	9.54 (0.0085)
	More than 5 and less than 10 years	220/284	77.46 (72.26–81.94)	1.92 (1.24–2.99)	
	Up to 5 years	34/94	36.17 (30.30–51.06)	Reference	
Contact type	Cattle + sheep	80/88	90.91 (83.08–95.32)	2.73 (1.53–4.85)	13.09 (0.0417)
	Cattle + buffalo + sheep + goat	173/226	76.55 (70.61–81.60)	2.27 (1.34–3.85)	
	Buffalo + sheep	67/93	72.04 (62.18–80.15)	2.16 (1.21–3.87)	
	Buffalo + goat	17/24	70.83 (50.83–85.08)	2.13 (0.97–4.66)	
	Cow only	40/61	65.57 (53.05–76.25)	1.97 (1.05–3.70)	
	Cattle + goat	12/21	57.14 (36.54–75.53)	1.71 (0.73–4.03)	
	Buffalo only	21/63	33.33 (22.95–45.63)	Ref	
Farm environment	Dry + hot	42/47	89.36 (77.40–95.37)	1.31 (0.85–2.02)	2.00 (0.3673)
	Dry + dusty	36/43	83.72 (70.03–91.88)	1.23 (0.77–1.94)	
	Dry	332/486	68.31 (64.04–72.29)	Reference	

### 3.4. Animal Contact Type‐Based Results

The seroprevalence of FMDV NSPs differed among the various animal‐contact categories. The highest seroprevalence was observed in animals kept with cattle and sheep (90.91%), animals kept with cattle, buffaloes, sheep, and goats (76.55%), buffaloes and sheep (72.04%), and buffaloes with goats (70.83%). The lowest seroprevalence (33.33%) was observed in herds with only buffaloes. Animals kept with cattle and sheep had significantly higher odds of NSP seropositivity (OR = 2.73, 95% CI: 1.53–4.85; *p* = 0.0417), suggesting that mixed‐species husbandry was significantly related to exposure to FMD (Table 1). The different groups of animals that were in contact with each other had significantly different mean OD values, according to an ANOVA (*F* = 23.77, *p*  < 0.001). Tukey’s post hoc test revealed significant differences between several contact groups.

The Tukey post hoc test identified significant differences in OD means among species contact groups. Spearman’s rank correlation (*ρ* = −0.222, *p*  < 0.0001) and linear regression both revealed a general trend of decreasing OD values with increasing species contact level, suggesting that OD values varied among the species contact groups. Overall, this dataset shows a negative correlation between the species contact level and OD, with higher encoded species contact levels associated with lower OD values.

### 3.5. Farm Environment‐Related Results

The seroprevalence of FMDV NSPs differed across farm environments. The highest seroprevalence was observed in animals reared in dry and hot conditions (89.36%; 42/47), followed by dry and dusty conditions with a seroprevalence of 83.72% (36/43), and dry conditions with a seroprevalence of 68.31% (332/486). The odds of having NSP seropositivity were higher in dry and hot environment (OR = 1.31) than in dry and dusty environments; however, this association was not statistically significant (*p* = 0.3673) (Table 1). There was a significant difference in mean OD among farm environments (ANOVA *F* = 7.62, *p* = 0.00054).

## 4. Discussion

Pakistan is an FMD‐endemic country with a large population of susceptible livestock [26]. FMD is a significant endemic disease in Pakistan, especially in areas where livestock are kept in large numbers and animals are moved frequently. In the present study, an overall positivity of 71.18% was observed for NSP antibodies among the cattle and buffaloes sampled from the nine livestock‐producing districts of Punjab, showing that the animals sampled in this study had a significant past exposure to FMDV. The high seropositivity rate aligns with FMD’s endemic nature in Pakistan and molecular studies showing ongoing circulation and evolution of FMDV in Pakistan [20, 21]. This is further reinforced by the epidemiological importance of the finding from outbreak investigations in Punjab, where animal movement, vaccination status, herd characteristics, and contact with neighboring livestock farms have been related as determinants to the occurrence of FMD [13].

The FMDV NSP 3ABC ELISA has a broad scope of applications for FMD surveillance since it detects antibodies to viral NSPs that are produced after the virus replicates, thus providing evidence of previous natural infection. In Pakistan, inactivated FMD vaccines are used, both locally produced and imported, as part of the provincial vaccination programmes. The use of purified vaccines allows for reliable DIVA application since it reduces contamination with NSP proteins, although this might sometimes lead to the detection of NSP antibodies in incompletely purified vaccines. The vaccination status of the animal populations sampled in the study could not be ascertained because the vaccination records available at the farms were incomplete, and some of the farmers were reluctant to provide accurate information regarding the previous vaccination history. Thus, the antibodies detected through 3ABC ELISA were interpreted as evidence of previous exposure to FMDV while acknowledging that residual NSP in incompletely purified vaccines may contribute to NSP seropositivity. Therefore, although the high NSP seroprevalence observed in this study is consistent with substantial exposure to FMDV, the available data could not determine the relative contribution of natural infection and vaccination. This is a limitation of the present study, and the reported NSP seroprevalence must be interpreted in light of the vaccination practices in the region. Recent experimental evidence has also shown that vaccination can affect humoral immune responses in cattle, increasing IgM responses and modulating the early immune response [27]. This demonstrates the complexity of interpreting antibody responses in vaccinated cattle and the importance of taking vaccination history into account when interpreting serological surveillance results.

The overall seropositivity in the present study was much higher than in an earlier 3ABC‐ELISA study carried out in Punjab. Nawaz et al. [28] investigated 900 cattle and buffaloes from Faisalabad, Khanewal, and Chakwal and found the overall NSP seroprevalence of 19.33% (23.09% in cattle and 16.04% in buffaloes). In this study, FMDV NSP seroprevalence was highest in Faisalabad, as in the previous study [28]. However, the higher estimate observed in the present study should not be directly compared to an increase in FMD prevalence over time, as the two studies differed in geographical coverage, sampling design, study populations, and local epidemiological circumstances. However, the repeated exposures measured in Faisalabad during both investigations are significant and suggest a continued high exposure pressure for FMDV in this large livestock production area. In line with this, there is also local evidence from the Faisalabad‐specific study of Nawaz et al. [29], where the overall seroprevalence was found as 28.40%, of which cattle showed 40.0% positivity compared to buffaloes 20.75%. However, despite the significant variation in seropositivity between studies, the exposure in Faisalabad is consistently higher, which could be due to livestock production, animal movement, and herd connectivity.

Ullah et al. [30] showed that there can be a high seropositivity for NSP in specific epidemiological settings. An overall NSP seroprevalence of around 67% in the northern border regions of Pakistan with marked differences between subregions (Khyber 46.5% and Swat 81.1%). A recent study in Pakistan from the Pakistan–Afghanistan border region further reinforces the idea that species‐related differences in FMDV exposure exist. Kabir et al. [31] performed both structural‐‐protein and NSP ELISAs on 800 cattle and buffaloes and reported 35.25% positivity overall, with 37% in cattle and 30% in buffalo. The authors also found spatial variation, herd size associations, and associations with other animal‐level factors. The results indicate that differences between cattle and buffaloes may be partly due to differences in exposure opportunities and livestock contact [31], not just differences in susceptibility.

The high seropositivity seen here is also similar to what has been seen in other parts of Pakistan. Khan et al. [32] recently reported active circulation of FMDV in cattle and buffaloes in Quetta, Balochistan, indicating that FMD is not limited to Punjab. In a buffalo‐based system, previous studies in the Landhi Dairy Colony, Karachi, have also shown significant epidemiological and economic impacts of FMD [12, 15]. Therefore, the high NSP positivity seen in Punjab should be viewed in the context of the national scenario of ongoing FMDV circulation in the country and not as a local phenomenon.

Another reason for the gap between the current estimate and the previous Punjab estimate may be changes in animal‐contact networks and livestock production systems. Highly interconnected livestock markets, dairy farms, breeding facilities, and rural production systems in Punjab could allow infected or previously exposed animals to move from one location to another. Ali et al. [13] conducted a detailed investigation of an FMD outbreak in Punjab and concluded that the introduction of animals with unknown vaccination status, lack of regular vaccination, animal‐broker visits, proximity to other livestock farms, and large herd size are important risk factors. Based on these observations, we suggest a plausible epidemiological explanation for the common exposure to NSP found in the current study.

The present results should also be interpreted in light of the diagnostic properties of NSP serology. Antibodies to FMDV NSPs are raised after viral replication and thus indicate past viral infection; therefore, the 3ABC ELISA is useful for surveillance in a vaccinated population [19, 25]. Furthermore, we could not generate reliable vaccination histories in this study because farm records were incomplete or farmers were unwilling to provide accurate information. Hence, the high seropositivity for NSP strongly suggests significant exposure to FMDV, but the proportion of exposure due to the natural infection and/or vaccine‐associated NSP responses cannot be determined with certainty.

### 4.1. Geographic Variation in Seropositivity

There was significant geographical variation among the nine study districts, with high seropositivity in Faisalabad, Bhakkar, and Bahawalnagar, while very low positivity was observed in Kasur. The magnitude of the geographical variation was epidemiologically significant, although the overall district‐wise association was not statistically significant. A previous study by Nawaz et al. [28] in Punjab also found significant geographical variation in NSP seropositivity, with Faisalabad having significantly higher NSP seropositivity than Khanewal and Chakwal. Faisalabad has repeatedly been identified as an area of high exposure, which may support continued transmission through local livestock production and livestock movements. So, it is improbable that geographical variation in the present study is due to differences of host susceptibility alone. Rather, it could be attributed to variations in livestock density, herd connectivity, animal movements, production systems, and/or market contacts.

Ali et al. [13] have offered an important explanation for this geographical heterogeneity through an outbreak investigation of Punjab. Proximity to other livestock farms was associated with higher odds of FMD outbreaks, and new animal introduction and contact with animal brokers were also considered important risk factors. Thus, the high seroprevalence of FMDV NSP in the specific districts in the current study may reflect more connected livestock movements and markets in these districts rather than differences in susceptibility. These findings have a special significance for the present study, as the districts with a high seropositivity level of NSP may possess potential high livestock trading intensity and farm‐animal market connectivity. Therefore, variations by district could reflect variations in opportunities for virus introduction and transmission and not intrinsic differences in susceptibility. The high seropositivity found in Faisalabad, Bhakkar, and Bahawalnagar should thus be interpreted as an indicator of possibly higher historical exposure pressure in these livestock networks.

The geographic heterogeneity found in the present study is similar to the latest evidence from Pakistan. Ullah et al. [30] found significant differences in NSP seroprevalence between the seven subregions of the northern border: from 46.5% in Khyber to 81.1% in Swat. Similarly, Kabir et al. [31] reported large variations in the seroprevalence of structural protein and NSPs in the border area between Pakistan and Afghanistan. This variation indicates that the likelihood of exposure to FMDV is likely to depend more on the local epidemiological circumstances rather than the administrative region of the country, such as livestock density, animal movement, herd structure, farming systems, and access to vaccination [30, 31].

Livestock density and livestock connectivity also illustrate the epidemiological importance, as shown by the study of Klein et al. [12] on the Landhi Dairy Colony: in this area, a high concentration of a large buffalo population in a highly connected production system provided favorable conditions for the persistence of FMD. Farooq et al. [15] then showed the significant economic impacts of FMD in the same production system. Although the production system in Landhi differs from the mainly rural systems studied here, the results from all of these showed that animal density, movement, and the relationship between production systems are relevant factors in FMD epidemiology in Pakistan.

The results of this study corroborate the general epidemiological picture of South Asia. A pooled prevalence of 43% was estimated from a systematic review and meta‐analysis of 73 FMD studies in India covering a total of 500,000 samples, with cattle having a higher prevalence compared to buffaloes (45% and 30%, respectively), and a significant geographical variation across different states [33]. Peng et al. [34] then further revealed in a global systematic review and meta‐analysis of swine FMDV that there was still significant geographic variability in FMD, which confirmed the persistent and heterogeneous global burden of FMD. The Indian analysis included different diagnostic methods and study designs, leaving it impossible to make any direct comparison with the current study; yet, the overall pattern of higher exposure seen in cattle compared to buffaloes offers regional support for this species pattern. The high geographical variation observed in India also highlights that exposure to FMD is not uniform but is greatly influenced by the local livestock production systems, animal density, FMD movement, and surveillance conditions.

FMD has also been shown to cluster geographically across neighboring livestock systems in South Asia. Using spatial and temporal analysis of FMD cases in cattle and buffaloes in Bangladesh, Rahman et al. [35] showed that FMD cases were geographically nonuniform and that some risk factors could be identified. This spatial variation is common in endemic FMD systems as the transmission of FMD does not occur evenly across a province but is dependent on factors such as livestock density, animal movement, herd connectivity, husbandry systems, and vaccination coverage.

### 4.2. Cattle and Buffaloes

The present study showed that the seropositivity of NSP was more prevalent in cattle than buffaloes, but it failed to reach statistical significance (OR = 1.14, 95% CI: 0.88–1.47; *p* = 0.3289). This association’s direction is in line with the previous investigations carried out in Punjab. Nawaz et al. [28] reported the prevalence of NSP as 23.09% in buffaloes and 16.04% in cattle, and another study conducted in Faisalabad by Nawaz et al. [29] showed 20.75% seropositivity in buffaloes and 40.0% in cattle. Similarly, the recent Pakistan–Afghanistan border study showed that the NSP positivity for cattle was higher as compared to buffaloes (37% vs. 30%) [31].

In all production systems of Pakistan, this is not the case. A seroprevalence of 46% of NSP was found among 636 large ruminants in peri‐urban dairy farms near Islamabad, with significantly higher seropositivity in buffaloes than in cattle [15]. That population had a high representation of buffaloes, mostly adult females, with buffaloes being significantly more likely to be NSP‐seropositive. This seemingly contradictory finding is epidemiologically relevant, as it indicates that differences in exposure to FMDV may be greatly influenced by the production system and animal management and not solely by differential susceptibility. Cattle and buffaloes from nine districts with varying livestock systems were included in the current study, while in the Islamabad study, peri‐urban dairy buffaloes prevailed. Because of these differences in animal behavior, herd structure, age distribution, production intent, and stocking density and contact, exposure between species may be different.

The greater seropositivity in cattle in the present study may be partly due to cattle having more opportunities to move, such as in breeding, dairy operations, purchase, and sale. However, buffaloes are still an epidemiologically important host in Pakistan. FMD transmission has been reported to be continuing at a high level, and major production losses have been reported in buffalo‐dominated dairy systems such as the Landhi Dairy Colony [12, 15]. Therefore, the reduced NSP positivity in buffaloes in the present study does not occur as evidence of buffaloes that have limited epidemiological importance. Instead, the findings suggest that the relative exposure of cattle and buffaloes could differ depending on local production and contact networks. Therefore, it is recommended that both species should be targeted in FMD control programmes in Punjab.

### 4.3. Sex‐Related Differences

Female animals were significantly more seropositive than males. In some endemic areas, similar sex differences in FMD seropositivity have been reported but have not been seen consistently across all geographical areas. Hence, the biological interpretation of this finding must be taken with a pinch of salt. A possible reason is the composition of the livestock sample in the present study. Most of the animals sampled were also females, reflecting the importance of dairy production in the study areas. Female dairy animals are kept longer for milk production and reproduction, while males can be sold or moved at younger ages. This means that females have a greater opportunity to be exposed to FMDV throughout their productive lives.

Animal demography and animal management characteristics have also been shown to be important in Pakistani studies. In the study by Nawaz et al. [29] in Punjab, age, species, and herd type were significantly associated with FMD seropositivity, while sex and pregnancy were not. Farooq et al. [15] likewise found no significant association between sex and NSP seropositivity in peri‐urban dairy farms near Islamabad. This difference is worth noting because it suggests that the strong sex association found in the present investigation may reflect the population sampling structure rather than a biological susceptibility difference between the sexes. The association between sex and seropositivity for FMD should be interpreted with caution. The sex distribution in this study was highly skewed. The present study showed a large predominance of females (534 females to 42 males), as it was carried out mainly in dairy production systems where females are preferentially selected for milk production. This should be considered when interpreting the OR. This imbalance may have reduced the accuracy of sex‐related comparisons and introduced sampling bias. Further research should include sex in addition to age, production purpose, species, and management system to test if sex is still independently associated with exposure to FMDV.

### 4.4. Seropositivity and Age

An epidemiological pattern was more evident with age. NSP positivity differed significantly between older animals (aged >5 years) and younger animals, and the odds of NSP seropositivity were significantly higher in animals older than 10 years. This observation is biologically plausible because NSP antibodies reflect previous exposure to FMDV, and older animals have had more time to be exposed to the virus.

It should be emphasized, however, that the age association is more likely to be a measure of accumulated exposure than to indicate that older animals are inherently more at risk. In endemic production systems, animals exposed to infected animals, contaminated environments, communal grazing areas, or livestock markets have more chances of coming in contact with infected animals when they are held longer. In addition, the earlier Punjab 3ABC‐ELISA study also found age to be a significant risk factor, supporting this interpretation [15, 28, 29]. The age gradient has implications for surveillance in practice. Older animals (such as breeding and dairy animals) that have undergone multiple production cycles may help gauge the historical FMDV circulation. Preventive measures should also ensure that young animals are protected since susceptible young stock can play a role in the transmission if there is inadequate population immunity.

The observed age‐associated increase in the present study agrees with previous studies from endemic areas. In Uganda, Dhikusooka et al. [36] showed persistent circulation of FMDV among cattle and found age‐related differences in epidemiology. More generally, Rodríguez‐Habibe et al. [37] highlighted that the immune system of animals infected with FMDV may differ from that of other age groups due to previous exposure and infection history, establishing a biological basis for cumulative serological differences among age groups.

### 4.5. Mixing of Species and Transmission of FMDV

One of the most relevant epidemiological associations from the present study is between mixed‐species husbandry and NSP positivity. Animals housed with both cattle and sheep were the most seropositive, while buffaloes housed only were the least seropositive. Animals housed with cattle and sheep also had a significantly higher likelihood of NSP seropositivity. This is biologically plausible as FMDV has a wide host range and different livestock species could play varying roles in disease spread. While there are only a few studies on mixing cattle–sheep, which directly tested this risk factor in Pakistan, there is local epidemiological evidence that highlights the importance of interherd and interspecies connectivity. The disease is FMDV with a wide host range, and sheep and goats are an integral part of the livestock production system in Punjab. A previous 3ABC‐ELISA survey of sheep and goats in Punjab reported an overall seroprevalence of 21% with significant regional variation [38]. Ali et al. [13] also showed that the movement of livestock, bringing animals from unknown vaccinated status, and animal‐broker visits, and farm proximity were also significant risk factors for FMD outbreaks in Punjab.

The higher proportion of positives noted in mixed‐species systems could reflect greater opportunities for interspecies spread rather than a specific biological effect of mixed‐species systems. This interpretation aligns with Paton et al. [18], who argued that understanding FMDV transmission should be approached across multiple scales, from individual animals and herds to larger livestock‐contact networks. As such, mixed‐species management should be regarded as part of the broader network of animal contacts involved in FMDV dissemination. Stenfeldt and Arzt [39] discussed the complexity of FMDV transmission and the ongoing uncertainties regarding the carrier state. Small ruminants are especially important because they may not become clinically apparent as quickly as cattle, allowing infected animals to move through the livestock network undetected. This is further supported by evidence from multispecies livestock systems. Bolortsetseg et al. [40] showed the potential benefit of multispecies serosurveillance for detection of FMDV exposure in livestock–wildlife interface populations, and Mkama et al. [41] reported FMDV exposure at these livestock–wildlife interfaces in Tanzania. Ploquin et al. [42] more recently showed that complex transmission patterns may result in multispecies livestock systems, especially when domestic animals are at risk of transmission from or to wildlife.

### 4.6. Serologic Reactivity and the Environmental Factors

We calculated means for the various environmental categories and found differences, but there was no statistically significant correlation between the environmental category and seropositivity for NSP. The results obtained should therefore be interpreted with caution. Local evidence suggests that environmental and seasonal factors may be factors that impact FMD occurrence. Khan et al. [43] examined a large number of serum samples (2511) from cattle and buffalo in rural areas of Khyber Pakhtunkhwa, where the seroprevalence for FMD was observed to be significantly seasonal, and farming systems, livestock movements, and meteorological conditions were suggested as potentially influencing factors. This could partly be due to the different geographical setting, climatic conditions, sampling duration, and environmental exposure classification used in the current study than that study. Broad categories in the present study, like “dry,” “dry and dusty,” and “dry and hot” may not reflect the quantitative differences in temperature, humidity, stocking density, ventilation, hygiene, water sharing, or animal movement. Environmental factors can then have an indirect effect on changing animal congregation and management and contact patterns instead of having a direct effect on FMDV exposure. Future studies should therefore gather quantitative data on stocking density, farm hygiene, shared water sources, livestock mobility, access to livestock markets, and vaccination coverage to see if environmental factors are still important when controlling for these potential confounding factors.

### 4.7. Vaccination and Disease Control

Based on the epidemiological pattern observed in the present study, animal‐ and population‐level factors sustain exposure to FMDV in Punjab. Age appears related to cumulative exposure, and mixed‐species husbandry provides more opportunities for interspecies transmission; geographical heterogeneity reflects differences in livestock connectivity and management. The overall conclusions of this study are consistent with the general principles of FMD control presented by Paton et al. [24], who highlighted the need for integration of surveillance, vaccination, movement management, and appropriate outbreak response for effective control. Paton et al. [18] also highlighted that FMDV transmission occurs at several epidemiological scales and that a single intervention is unlikely to effectively control it.

The local evidence is of particular importance in this regard. Ali et al. [13] identified the following risk factors for FMD outbreaks in Punjab: irregular vaccination, introduction of animals with unknown vaccination status, animal‐broker visits, farm proximity, and herd size. Likewise, Farooq et al. [44] reported substantial economic losses due to FMD in an important dairy system of Pakistan. This suggests that vaccination should be used alongside movement restrictions, quarantine for newly introduced animals, and checks on vaccination status and farm biosecurity.

Recent studies on FMD vaccination also show that there is scope to enhance vaccine‐induced protection by modulation of host immune responses. Kim et al. [45] showed that the use of D‐galacto‐D‐mannan as a mucosal stimulator could improve systemic immune responses to FMD vaccination. While this cannot be directly extrapolated from the current cross‐sectional serological survey, it gives an important perspective for future FMD control programmes. In addition to sufficient vaccination coverage, surveillance, movement control, quarantine, and farm‐level biosecurity, boosting vaccine‐induced immunity in endemic regions like Punjab may offer a more holistic strategy for reducing the spread of FMD.

Further evidence of molecular data from Pakistan suggests the need for ongoing surveillance. Jamal et al. [21] reported the presence of the O/ME‐SA/Ind‐2001e sublineage in Pakistan and the emergence of new serotype O sublineages in 2012–2021 by Jamal et al. [20]. Ideally, epidemiological surveillance using NSP serology should be supplemented by molecular surveillance for the identification of viruses in circulation in different production systems and for strain selection of vaccine. On the practical level, the present findings indicate that vaccination should be continued, that vaccination status should be checked before introduction of animals at the farm, that the newly introduced animals should be kept under quarantine, that biosecurity measures should be improved at the farms, that movement of livestock should be managed, and that increased surveillance should be undertaken in the districts that showed higher exposure.

### 4.8. Limitations and Interpretation

A limitation of the present study is the lack of reliable vaccination histories. While 3ABC ELISA can detect previously exposed animals, results must be interpreted in light of vaccine purity and vaccination history [19, 25]. Observed seropositivity should be interpreted as evidence of high prior exposure and not as an estimate of the prevalence of active infection. Another limitation of convenience sampling was that the observed prevalence cannot be extrapolated to the total cattle and buffalo population of Punjab. Despite this, the study included 9 important livestock‐producing districts and evaluated several animal‐ and farm‐level factors, providing useful epidemiological evidence. The findings were consistent with a few other studies conducted in Pakistan, which also highlighted the geographical variation, differences between cattle and buffaloes, and age‐related exposure; hence the relevance of the findings.

In general, the comparison with Pakistani literature reveals that there is no contradiction between the studies, as the apparent differences are not always contradictory. Rather, they emphasize the diverse epidemiology of FMD in Pakistan. Observed seroprevalence can be significantly affected by differences in study design, diagnostic methods, animal demographics, geographical setting, livestock density, production systems and animal movement patterns, amongst other factors, and vaccination status. The present study, in line with previous studies conducted in Pakistan, showed that FMDV NSP seropositivity was age‐dependent and also varied geographically, while other production systems reported exposure with different species and sex, indicating that FMDV NSP seropositivity is very context dependent. This factor should be taken into account when interpreting serological estimates and formulating control programmes at the regional level.

Overall, the current study contributes multidistrict serological data to the available literature of the outbreak and molecular data of FMD in Pakistan. The high NSP positivity, geographical heterogeneity, seropositivity increasing with age, and higher positivity in cattle, and association with mixed‐species husbandry systems suggest high‐level historical exposure to FMDV in interconnected livestock populations in Punjab. These results indicate that a risk‐based approach to FMD control, incorporating vaccination with animal‐movement management, quarantine, farm biosecurity, and ongoing epidemiological and molecular surveillance, is important.

## 5. Conclusion

The study presents crucial epidemiological findings regarding FMDV distribution in Punjab, Pakistan. This study demonstrates that advanced animal age alongside female animals and a larger proportion of cattle populations lead to increased FMDV NSP seroprevalence, which highlights critical risk factors demanding specific preventive measures. The development of disease becomes worse because farms combine different species together and have weak biosecurity measures that require improved control strategies. The implementation of robust biosecurity measures together with vaccination programs has strong potential to reduce FMD occurrence in Pakistan.

## Funding

This work was funded by the Science and Technology Projects of Rikaze Autonomous Region, China (Grant RKZ2025ZD‐009), the International Science and Technology Innovation Program of Chinese Academy of Agricultural Sciences (Grants CAASTIP‐2026‐05, CAAS‐ZDRW202607, and CAAS‐ASTIP‐2021‐LVRI), the Foreign Expert Project (Grant H20250528), the Deanship of Scientific Research, Vice Presidency for Graduate Studies and Scientific Research, King Faisal University, Al‐Ahsa, Saudi Arabia (Grant KFU264629), the Major Science and Technology Project of Gansu Province (Grants 24ZD13NA008, 23ZDNA007, and 22ZD6NA001), and the NBCITS (Grant CARS‐37).

## Disclosure

The authors have nothing to report.

## Ethics Statement

The research was conducted as part of a standard veterinary field survey of FMD and in accordance with the approval from Directorate of Graduate Studies, University of Agriculture, Faisalabad, Pakistan.

## Conflicts of Interest

The authors declare no conflicts of interest.

## Data Availability

All data associated with this study are included in this manuscript.
